# Protein kinase Ime2 is associated with mycelial growth, conidiation, osmoregulation, and pathogenicity in *Fusarium oxysporum*

**DOI:** 10.1007/s00203-022-02964-0

**Published:** 2022-07-05

**Authors:** Jiling Xiao, Yi Zhang, Ke Yang, Yanying Tang, Lin Wei, Erming Liu, Zhihuai Liang

**Affiliations:** 1 College of Plant Protection, Hunan Agricultural University, Changsha, 410125 China; 2 Hunan Agricultural Biotechnology Research Institute, Changsha, 410125 China; 3 Hunan Plant Protection Institute, Changsha, 410125 China; 4 Hunan Rice Research Institute, Changsha, 410125 China

**Keywords:** *Fusarium oxysporum*, MAPKs, Protein kinase, Ime2

## Abstract

*Fusarium oxysporum* f.sp. *niveum* is one of the most serious diseases impairing watermelon yield and quality. Inducer of meiosis 2 (Ime2) is the founding member of a family of serine/threonine protein kinases and plays important roles in yeasts and other filamentous fungi. In this study, we analyzed the functions of *Fo*Ime2, the ortholog of *Saccharomyces cerevisiae* Ime2 in *F. oxysporum* f.sp. *niveum*. The *FoIme2*-deleted mutants exhibited obvious morphological abnormalities, including slower vegetative growth, more branches in the edge hyphae and a reduction in conidia production. Compared to the wild type, the mutants were hypersensitive to the osmotic stressor NaCl but were more insensitive to the membrane stressor SDS. The deletion of *FoIme2* also caused a reduction in pathogenicity. Transcriptional analysis revealed that *Fo*Ime2 acts downstream of *Fo*Opy2 which is an upstream sensor of the MAPK kinase cascade. These results indicate that *Fo*Ime2 is important in the development and pathogenicity of *F. oxysporum,* and provide new insight for the analysis of the pathogenic mechanism of *F. oxysporum.*

## Introduction

The soil-borne, asexual fungus *Fusarium oxysporum* Schlechtend.: Fr. f. sp. *niveum* (E.F. Sm.) W.C. Snyder & H.N. Hans (Fon), is the dominant pathogen of Fusarium wilt on watermelon (*Citrullus lanatus*) worldwide, causing adverse impacts on watermelon yield and quality(Hudson et al. 2021).To adapt to saprophytic and parasitic environments, *F. oxysporum* needs to sense and respond to numerous stimuli from their environment, including their host organisms(Kou and Naqvi 2016). Molecular perception of these stimuli is regulated by a large number of different protein kinase channels, of which mitogen-activated protein kinase (MAPK) cascades are among the most important and highly conserved members (Vandermeulen and Cullen 2020; Wu et al. 2019). Most ascomycete fungi including *F. oxysporum* possess only three MAPKs, Fmk1, Mpk1, and Hog1, which are orthologous to yeast Fus3/Kss1, Mpk1, and Hog1, respectively (Martinez-Soto and Ruiz-Herrera 2017; Pareek and Rajam 2017; Segorbe et al. 2017; Turra et al. 2014). Moreover, the inducer of meiosis 2 (Ime2) homolog pathway is believed to be a new MAPK pathway in several fungi (Xie et al. 2019). Ime2 belongs to the serine/threonine protein kinase family and is conserved in all eukaryotes. A major characteristic of these kinases is their similarity in the N-terminal region, which includes a TXY motif, typically found in the activation loop of MAPKs (Irniger 2011). Ime2 was first identified as a gene expressed exclusively during meiosis in *S. cerevisiae* (Smith and Mitchell 1989; Schindler and Winter 2006). Subsequent studies demonstrated that it also plays key roles in sporulation and pseudohyphal growth in yeast (Sari et al. 2008; Strudwick et al. 2010). During the last few years, Ime2-related protein kinases from various fungal species have been studied. Surprisingly, these protein kinases exhibited striking diversity in their cellular functions. In *Aspergillus nidulans*, the Ime2 homologous protein IMEB does not participate in meiosis, but functions as an inhibitor of sexual development in the presence of light. IMEB also promotes asexual reproduction, and regulates the production of secondary metabolites in *A. nidulans* (Bayram et al. 2009). For *Neurospora crassa*, Ime2 is needed for the inhibition of protoperithecia formation in response to the availability of sufficient nitrogen (Hutchison and Glass 2010). Ime2 is also reported to be negative regulator of the cell death pathway to specifically regulate nonself recognition and cell death in *N. crassa* (Hutchison et al. 2012). The analysis of the Ime2 homolog Crk1 in the phylum of the basidiomycete suggests that this protein kinase is involved in the regulation of mating. However, it negatively affects mating in *Cryptococcus neoformans* but promotes mating by regulating the expression of the *prf1* gene in *Ustilago maydis* (Garrido et al. 2004; Liu and Shen 2011). In *Trichoderma reesei*, Ime2 is involved in the degradation process of the plant cell wall: deletion of the Ime2 homologous genes results in significantly upregulated expression of cellulase *cbh1*, *cbh2*, and *egl1* (Chen et al. 2015). In the nematode predatory fungus *Arthrobotrys oligospora*, the absence of Ime2 results in slow growth, reduced sporulation, loose cell wall structure, reduced trapping structure, and lower nematode capture quantity than that of the wild type (Xie et al. 2019). Thus, Ime2-related kinases exhibit an amazing variety of functions in controlling cellular processes in fungi.

To date, the functions of the protein kinase Ime2 in *F. oxysporum* have not been reported. In this study, we identified homologs of *S. cerevisiae* Ime2 in *F. oxysporum*. The functionalities of *Fo*Ime2 in *F. oxysporum* were investigated by constructing deletion mutants. Compared to the wild type, the FoIme2-deleted mutants exhibited obviously decreased mycelial growth and conidiation production, and more branches in the edge hyphae. The mutants were hypersensitive to the osmotic stressor NaCl but more insensitive to the membrane stressor SDS. The deletion of FoIme2 also caused a reduction in pathogenicity. Our results suggest that *Fo*Ime2 is a general regulator of morphogenesis and virulence in *F. oxysporum.*

## Materials and methods

### Fungal strains and culture conditions

The wild type strain *F. oxysporum* f.sp. *niveum* races 1 (Fon-1) was isolated from the roots of symptomatic field-grown plants in Changsha, China. Race determination was performed on watermelon differentials Black Diamond, Charleston Gray,and Calhoun Gray (Fulton et al. 2021). Fon-1 was used as the parental strain for the transformation experiments and was maintained as a stock culture in our lab. Potato dextrose agar (PDA) was used as the routine medium for wild type cultures. PDA supplemented with 200 μg/ml neomycin (Solarbio, Beijing, China) was used for subculturing deletion mutants. Potato dextrose broth (PDB) was used for fungal mycelia and conidia production or DNA and RNA extraction. All cultures were grown at 28 °C. All fungal isolates were purified as single spore cultures and stored at − 80 °C in a 30% (*v*/*v*) glycerol solution.

### Sequence analysis of ***FoIme2*** in *F. oxysporum*

The FoIme2 gene of *F. oxysporum* was originally identified through the homology searches of the *F. oxysporum* genome sequence, using the BLASTX algorithm and the sequences *S. cerevisiae* Ime2 protein sequence as the query.

## Construction of *FoIme2*-deleted mutants

Target gene deletion was carried out by replacing the *FoIme2* gene (GenBank number: MAMH01000468.) with a neomycin resistance marker cassette using the split-marker methodology. The vector pKN- *FoIme2*-KO was created to generate two overlapping gene deletion constructs. All primers are listed in Table 1. A 1597 bp upstream fragment and a 1365 bp downstream fragment of the target gene were amplified from the genomic DNA of the WT strain with the primer pairs IU-*Sac*I-F /IU-*Not*I-R and ID-*Spe*I-F/ID-*Apa*I-R, respectively. The upstream fragment was digested using the restriction enzymes *Sac*I/*Not*I and inserted into the restriction sites *Sac*I/*Not*I of pKN vector containing *neo* (encoding neomycin phosphotransferase) to produce the vector pKN-*FoIme2*-FS. Next, the downstream fragment was digested using the restriction enzymes *Spe*I/*Apa*I and inserted into the restriction sites *Spe*I/*Apa*I of pKN to produce vector pKN- *FoIme2*-KO. The first fragment contained the upstream sequence of *FoIme2,* and the 3ʹ end (approximately 75%) of the neomycin cassette, was obtained by amplification with the specific primer pair IU-*Sac*I-F/Neo-R. The second fragment contained the terminator region of the target gene and the 5ʹ end (approximately 75%) of the neomycin cassette was obtained by amplification with the specific primer pair Neo-F/ID-*Apa*I-R.

**Table 1:** All primers used in the study

Primers	Sequence(5′–3′)	PCR purpose
IU- SacI-F	CGAGCTCCTTCGCTTGTCTACGACAGTTT	Amplify the left homologous arm of *foime2*
IU- NotI-R	ATAAGAATGCGGCCGCATGTGGCGGCAGTAAAGG	
ID-SpeI-F	GACTAGTCGTCTTGACATAATGTTACGATTAC	Amplify the right homologous arm of *foime2*
ID-ApaI-R	ATTGGGCCC GCCTAAGGTAAGCGTCGTG	
Neo-F	AGCCAACGCTATGTCCTGATAGCGGTC	Amplify the *neo*-cassettes and downstream
Neo-R	GTCAAGACCGACCTGTCCGGTGCCCTG	Amplify the upstream and *neo*-cassettes
Neo-check-F	ACCTATATCTGCGTGTTGCCTGTA	Amplify the 1665 bp fragment of *Neo*
Neo-check-R	TGATTGGCGGCAGGAATATGATG	
Ime2-check-F	GGTAAGTCAATACGATGTGCAC	Amplify the 1116 bp fragment of *foime2*
Ime2-check-R	AGGTCAGTGCTCTCAGAACGT	
PrF	CTCACCTGTCGACGCCTCTG	Amplify the 523 bp probe for Southern blot
PrR	CGATATTAACGAAAAGGAGA	
Opy2-F	AGCGAGAGCTGCCGATATACC	Detecting the expression of *FoOpy2*
Opy2-R	GCGAAAATTACTACAATGCTAGCGATGA	
Sho1-F	TGGACGGATACAACAAAATAGCG	Detecting the expression of *Fosho1*
Sho1-R	GCCAACAATCAGGCATAGACAG	
Ste50-F	GACCGTTGAGGAATGTGCTG	Detecting the expression of *FoSte50*
Ste50-R	GAGGATCGTTAGGCGATGTC	
Ste12-F	CAGAATATGTCAAGGCATGGAAC	Detecting the expression of *FoSte12*
Ste12-R	GTTGGGAGCAGGAACAGAGC	
C1-F	CCCCTCGTGCTTCACAACAA	Detecting the expression of *FOXG_12855*
C1-R	CTCAGTGCTCTTCCAGGTGCTAC	
C2-F	CGACACGACCAAGAAGACCAT	Detecting the expression of *FOXG_13111*
C2-R	TGCATCCAAAGTAGGCACAGTAG	
C3-F	GACTCTGACAGCTCCGGTACTTC	Detecting the expression of *FOXG_16880*
C3-R	CCGATCATGCCAACCTTCTT	
C4-F	GTATCACCGTCTGGGGCGTAT	Detecting the expression of *FOXG_14504*
C4-R	AGAGCGTTGACAACAGCAGTGTA	
Actin-F	ATGTCACCACCTTCAACTCCA	Detecting the expression of *Actin*
Actin-R	CTCTCGTCGTACTCCTGCTT	
EF-F	CATCGGCCACGTCGACTCT	Detecting the expression of *EF2*
EF-R	AGAACCCAGGCGTACTTGAA	

“—” Represents the restriction site or guard base

PEG-mediated transformation was performed as described previously (Di Pietro and Roncero 1998). The transformants were cultured in PDA plates supplemented with 200 μg/ml neomycin and grown at 28 °C. To identify the gene-deleted mutants, the resulting transformants were screened by PCR with the primer pairs Neo-check-F/Neo-check-R, and Ime2-check-F/Ime2-check-R (Table 1) which were used to identify the neomycin resistance and *FoIme2* gene, respectively. The deletion mutants were further confirmed by Southern blot analysis of *Eag*I-digested genomic DNA using a PrF/PrR (Table 1) PCR-amplified 523 bp fragment as the probe.

### Mycelial growth and conidiation

To determine the mycelium growth rate, mycelial plugs of wild-type or mutant strain (5 mm diameter) from the edge of a 5-day-old colony were transferred to potato PDA containing the corresponding antibiotics, and grown in the dark at 28 °C. Each strain was represented by three replicate plates. After 3 days, the colony diameter in each plate was measured and the average diameter was calculated in each group. These experiments were repeated four times.

For the conidiation assay, three mycelial plugs (5 mm diameter) from the edge of a 5-day-old colony were transferred to a flask containing 50 ml of PDB (150 rpm, 28 °C). After 4 days, microconidia were counted with a hemocytometer. Each strain was represented by three replicate flasks. The spores were imaged through a microscope, to detect changes in the conidium morphology. The experiment was repeated three times.

Synthetic low-nutrient agar medium (SNA), containing (in *w*/*v*) 0.1% KH_2_PO_4_, 0.1% KNO_3_, 0.05% MgSO_4_ ·7H_2_O, 0.05% KCl, 0.02% glucose, 0.02% sucrose, and 2% agar, was used to induce macroconidia and chlamydospores. Agar blocks (5 mm in diameter) carrying mycelia of wild-type or mutant strains were inoculated onto SNA and incubated at 28 °C for 7 days under continuous black–blue light. The conidiation was observed with a light microscope (Nguyen et al. 2019).

### Sensitivity to compounds causing osmotic, cell wall, cytoplasm membrane stress or oxidative stress

Mycelial plugs (5 mm diameter) taken from the edge of a 5-day-old colony were grown in the dark at 28 °C in 5-cm-diameter Petri plates (each plate contains one Fon-1 wild type plug, one *ΔFoIme2*-2 plug and one *ΔFoIme2*-19 plug) containing PDA amended with different concentrations of NaCl, KCl, SDS, Congo red (CR), and H_2_O_2_ for 3 days. The diameter of the colonies were measured every 24 h. The percentage of mycelial radial growth inhibition (RGI) was calculated using the formula RGI = [(*C*–*N*)/(*C*–5)] × 100%, where *C* is the colony diameter of the control and *N* is that of a treatment. Each combination of strain and stressor was represented by three replicate plates, and the experiment was repeated three times.

### RNA extraction and quantitative real-time PCR analysis

Total RNA was extracted from the mycelia of each sample using RNA-easy™ Isolation Reagent (Vazyme Biotech, Nanjing, China). One microgram of each RNA sample was used for reverse transcription with a HiScript^®^ II 1st Strand cDNA Synthesis Kit (+ gDNAwiper) (Vazyme Biotech, Nanjing, China). The expression levels of *FoOpy2*, *FoSho1*, *FoSte50*, *FoSte12* and four cellulase genes (*FOXG_12855*; *FOXG_13111*; *FOXG_16880*; *FOXG_14504*) under induction in PDB medium were determined by quantitative real-time reverse transcriptase PCR (qRT–PCR) with the primers listed in Table 1. For each sample, the *F. oxysporum Actin* and *EF2* genes were used as reference sequences. The analysis of relative gene expression levels using the qRT–PCR data was performed according to the 2^−ΔΔCT^ method described previously (Livak and Schmittgen 2001). The experiment was repeated three times.

### Plant material and inoculation

“Zaojia 8424” [*Citrullus lanatus* (Thunb.) Matsum. and Nakai., Xinjiang Academy of Agricultural Sciences, China], a commercial watermelon variety that is susceptible to race 1 of FON, was used for this study. Watermelon germinating seeds were planted in soils that were sterilized and inoculated with conidial suspensions (conidial content of 1 × 10^4^ conidia/g) and maintained in a growth chamber (14 h light at 28 °C/10 h dark at 24 °C). The disease severity of each plant was evaluated based on external yellowing and wilting scores as follows: 0, no symptoms; 1, slight yellowing of cotyledons; 2, yellowing and wilting of cotyledons; 3, yellowing of cotyledons and true leaves; 4, yellowing and wilting of all leaves except the heart; and 5, wilting of all leaves or plant death. The disease index (DI) was calculated using the formula DI = Σ(Xi*n)/5*N × 100%, where *N* is the number of investigative watermelon seedlings for each strain; Xi is the level of wilting symptoms (*i* = 1,2,…5); and n is the number of watermelon seedlings with the same wilting symptoms of each strain symptoms. Each strain was represented by thirty replicate plates, and the experiment was repeated 3 times.

## Results

### Identification of *ime2* from *F. oxysporum*

*FoIme2* (GenBank number: MAMH01000468.) was originally predicted by BLAST analysis of the model fungi *S. cerevisiae* and *N. crassa.* Ime2 proteins against the *F. oxysporum* f. sp. *niveum* genome sequence (available at https://www.ncbi.nlm.nih.gov/genome/707?genome_assembly_id=280512). The full *FoIme2* gene in *F. oxysporum* is 2412 bp long, contains two 57 bp introns and encodes a protein with 765 amino acids. The search for conserved domains against the NCBI Conserved Domain program revealed that *Fo*Ime2 contains the conserved catalytic domain of S_TKc associated serine/threonine kinases (residues 24–352).

### Deletion of *FoIme2* in* F. oxysporum*

**Fig. 1:**
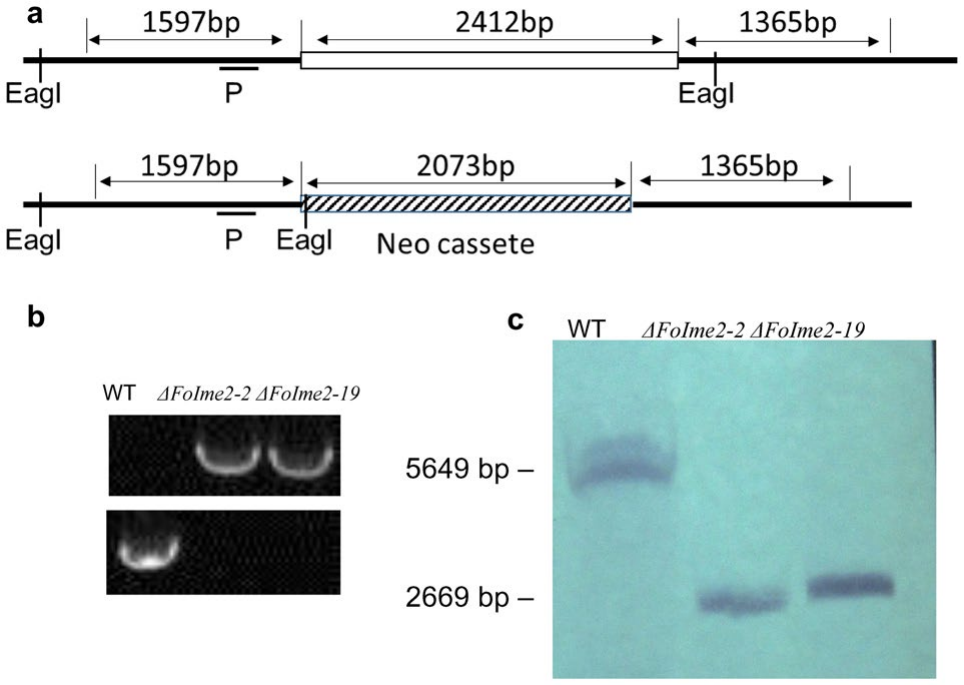
Schematic representation of the gene deletion vector strategy and identification of *Foime2* deletion mutants. **a** Schematic representation of the gene deletion vector strategy. **b** Positive transformants were verified by PCR amplification*.* **c** The Δ*FoIme2* mutants were confirmed by blotting analysis

To understand the biological functions of *Fo*Ime2 in *F. oxysporum,* we generated gene-deletion mutants using a fusion PCR-based deletion strategy (Fig. 1a). After PCR screening (Fig. 1b), two stable mutants were used for Southern blot analysis.

Southern blot analysis used a 523 bp fragment located upstream of *FoIme2* as the probe. When probed, the deletion mutants produced an anticipated 2669 bp band, whereas the wild-type strain produced an anticipated 5649 bp band (Fig. 1c). The genetically stable gene deletion transformants for *F. oxysporum* wild-type strains were named *ΔFoIme2*-2 and *ΔFoIme2*-19*,* respectively.

### Deletion of *FoIme2* affects mycelium growth and conidial yield

**Fig. 2:**
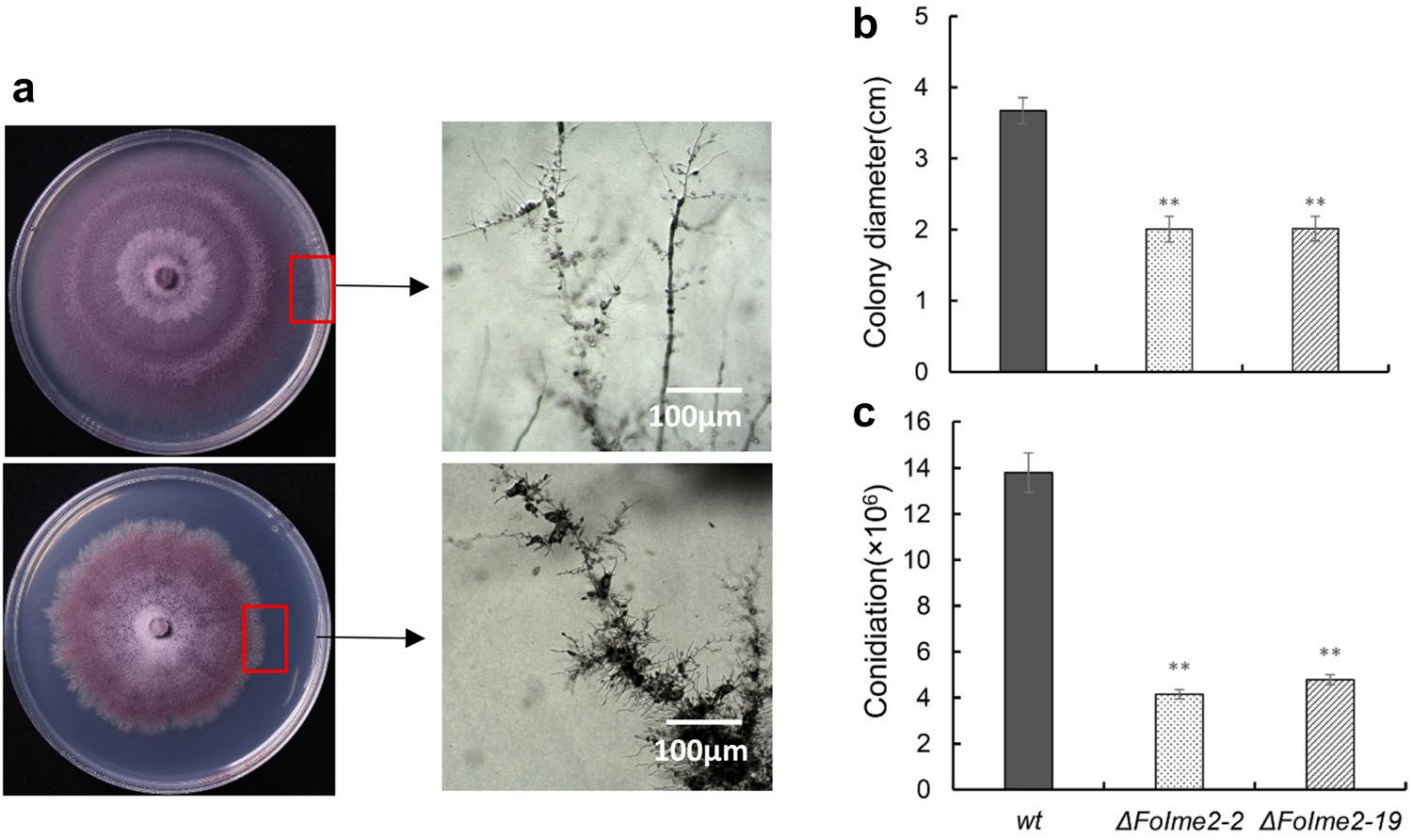
Comparison of mycelial growth, morphology, and septum formation between wild type and *∆Foime2* strains. **a** Colony morphology and hyphae of WT and 1Aoime2 mutant strains incubated on PDA medium for 10 days at 28 °C. **b** Colony diameters of WT and mutants incubated on PDA for 3 days. **c** Significantly reduced numbers of microconidia were shown by mutants in PDB

Significant reductions were observed in the growth of two independent mutants (*ΔFoIme2-2* and *ΔFoIme2-19*) in comparison to the wild-type (Fon-1) colonies. Furthermore, when incubated on PDA at 28 °C for a long time (more than 10 days), the edge hyphae of the mutants colonies were more likely to produce many more branches than those of WT colonies (Fig. 2a). The macroconidia produced by the mutants were not different in morphology from those produced by Fon-1. However, a significantly reduced number of microconidia was produced by mutants in PDB (Fig. 2c).

### *FoIme2* contributes to the stress response in *F. oxysporum*

**Fig. 3:**
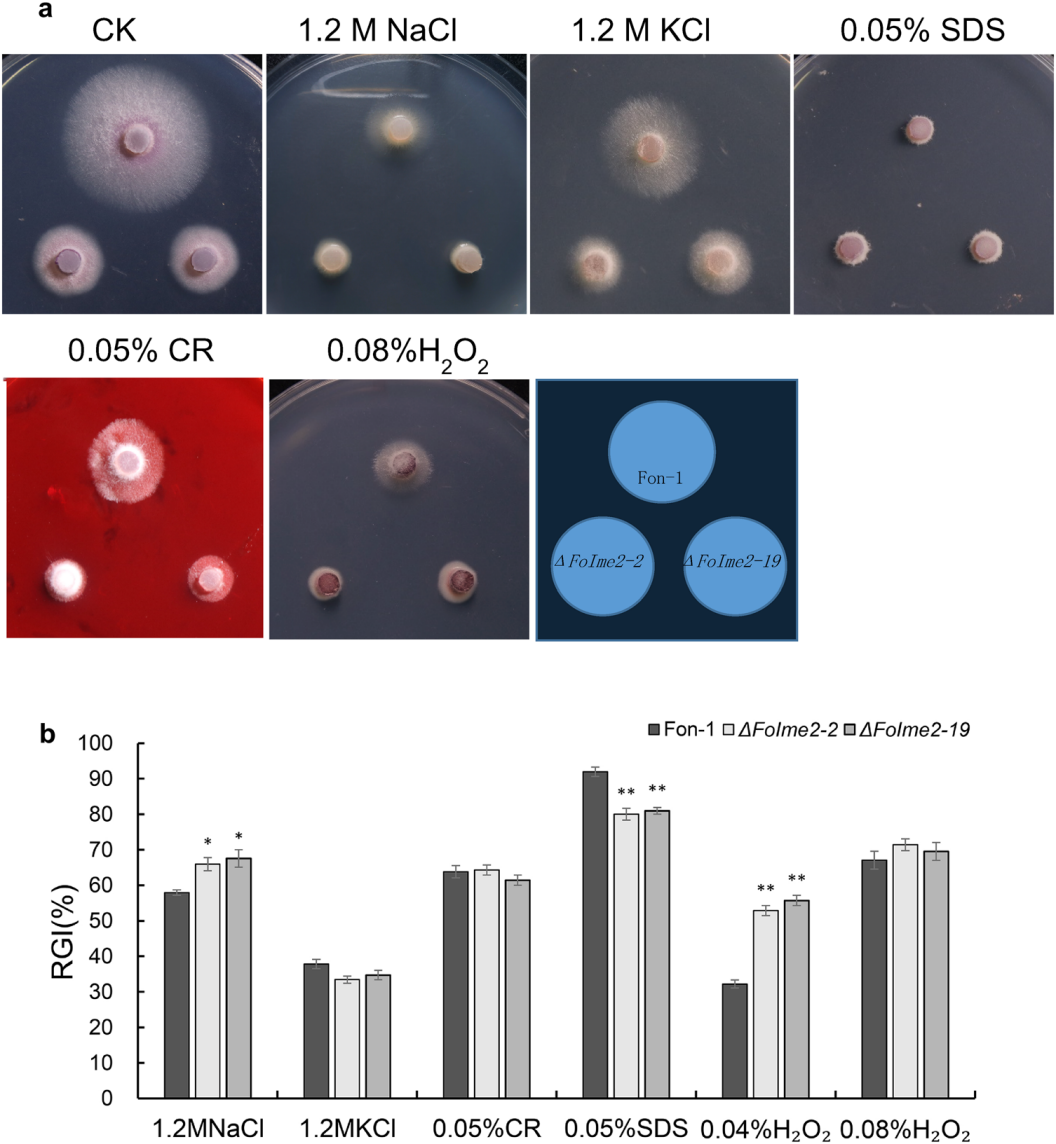
Vegetative growth of Fon-1, *ΔFoIme2-2* and *ΔFoIme2-19* strains under different stressors. **a** The Fon-1, *ΔFoIme2-2* and *ΔFoIme2-19* strains were cultured on PDA,and PDA medium with 1.2 M NaCl, 1.2 M KCl 0.05% SDS, 0.05% Congo red, and 0.08%H_2_O_2_. **b** The relative inhibition rate after 3 days of incubation was statistically analyzed using colony diameter as an indicator. The average and standard deviation of each set of data were calculated based on three iterations. Asterisks indicate a significant difference (“*” means *P* < 0.05; “**” means *P* < 0.01)

The *FoIme2* mutants displayed decreased resistance to extracellular osmotic (NaCl) stress and increased resistance to membrane (SDS) stress compared to the wild-type strain. However, no differences in the tolerance to intracellular osmotic (KCl), cell wall (CR), and oxidative (0.08%H_2_O_2_) stress were identified between the mutants and the wild-type strain (Fig. 3a, b).

### *FoIme2 *is essential for the full virulence of *F. oxysporum.*

**Fig. 4:**
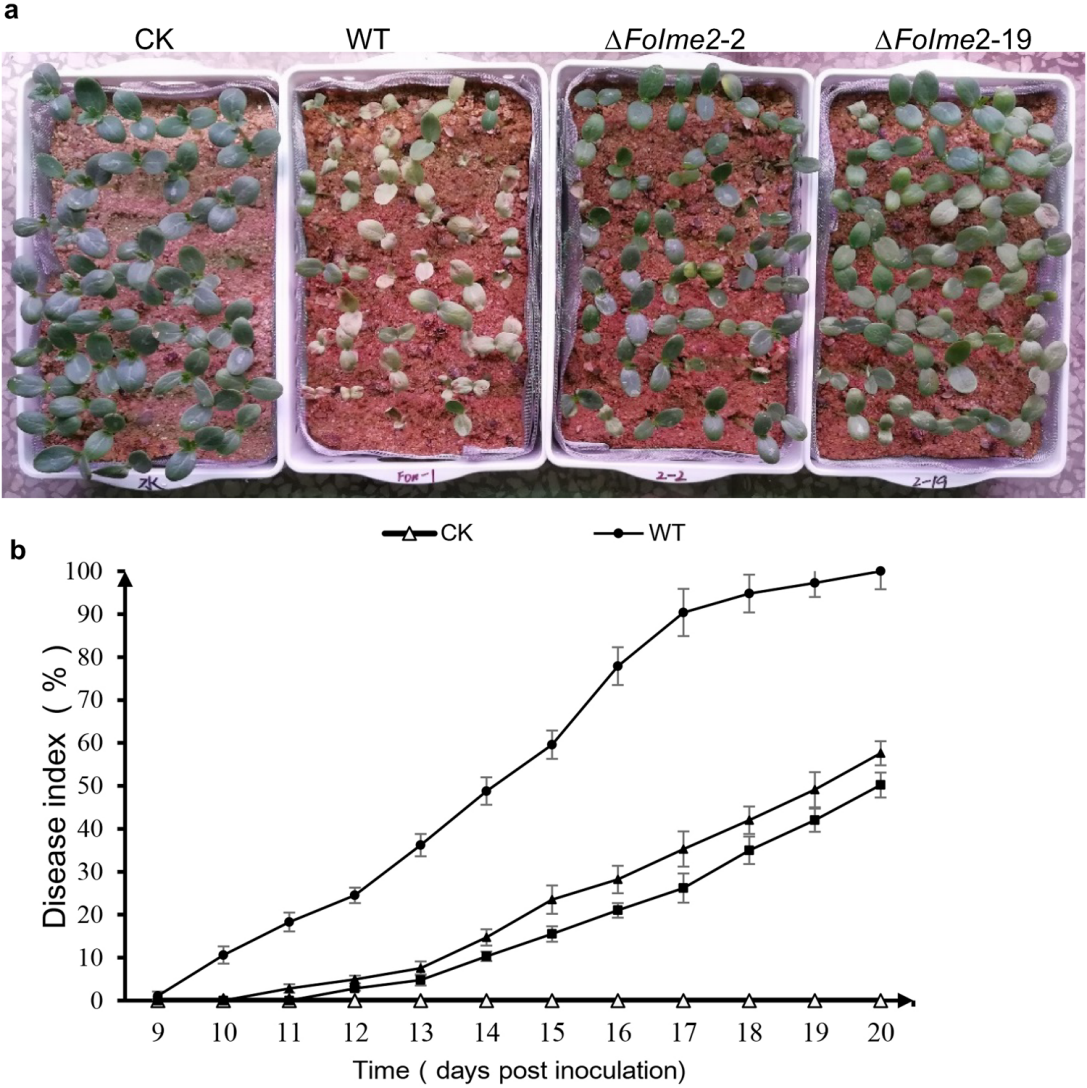
FoIme2 is required for of *Fusarium oxysporum* virulence on watermelon plants. **a** Watermelon seedlings were cultivated in soil with conidial suspensions of Fon-1, *ΔFoIme2-2* and *ΔFoIme2-19* and photographed at 14 dpi. **b** Disease index of watermelon 9–20 days after seeding

The virulence of the *FoIme2-*deleted mutants was determined by plant infection assays. Watermelon planted in soils inoculated the with wild type or mutant strains all showed characteristic wilt symptoms. However, the mutants exhibited impaired pathogenicity due to a significant delays in initial symptom appearance (Fig. 4a, b). The results demonstrated that *Fo*Ime2 is essential for the potency of pathogenicity in *F. oxysporum*.

## Discussion

In *S. cerevisiae*, Ime2 was first identified as a gene expressed exclusively during meiosis which was initiated by the transcriptional activator Ime1 (Smith and Mitchell 1989). The sexual reproduction period of *F. oxysporum* is currently unknown and the homologous genes of *ime1* are absent in the *F. oxysporum* genome. In this study, we identified the function of the protein kinase Ime2 in *F. oxysporum,* which regulates mycelium growth, microconidia development, extracellular osmotic and membrane integrity. These results suggested that Ime2 may play more roles in addition to meiosis in fungi whose sexual reproduction is not important for their life-cycle stage.

Significant reductions in the growth of two independent *FoIme2-deleted* mutants was observed. The hyphal morphology of the mutants was different from that of the wild type. This result agrees with what has been observed with the ime2 mutants of several filamentous fungi. In *Nomuraea rileyi*, *A. oligospora.*, and *U. maydis*, the deletion of the *ime2* homologous gene severely affected the growth rate of the strain and the filamentous growth (Garrido et al. 2004; Li 2018; Xie et al. 2019). The phenotype of imeB mutants in *A. nidulans* also showed a reduction in vegetative growth (Bayram et al. 2009).

**Fig. 5:**
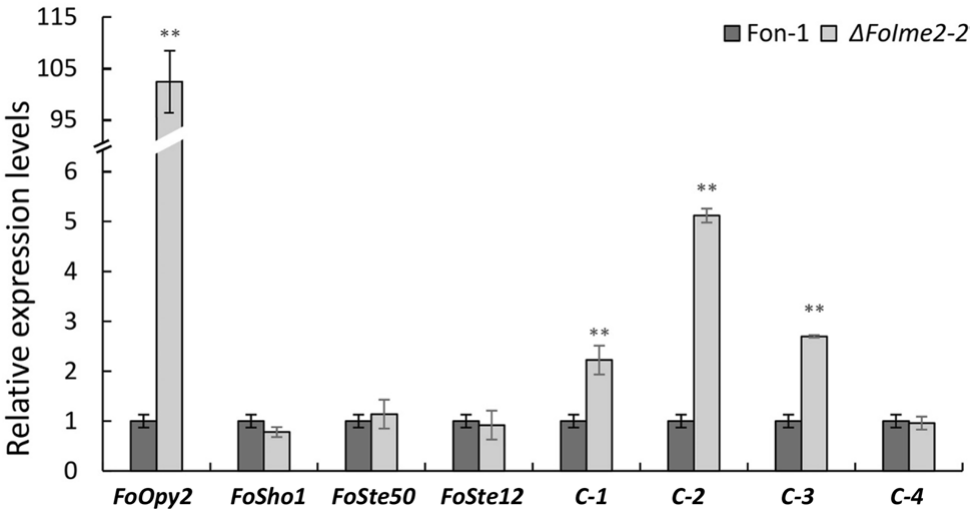
The relative expression levels of *FoOpy2, FoSho1, FoSte50, FoSte12* and four cellulase genes in the Fon-1 and *ΔFoIme2*-mutants were determined by quantitative real-time polymerase chain reaction (qRT-PCR). *C-1*: *FOXG_12855*;* C-2*: *FOXG_13111*; *C-3*: *FOXG_16880*; *C-4*: *FOXG_14504*). Asterisks indicate a significant difference (“*” means *P* < 0.05; “**” means *P* < 0.01)

A previous study showed that the filamentation of *F. oxysporum* was regulated by Fmk1 MAPK cascades (Di Pietro et al. 2001; Segorbe et al. 2017). *Fo*Opy2, *Fo*Sho1, *Fo*Ste50, and *Fo*Ste12 are participants in Fmk1 MAPK cascades. The transmembrane proteins Opy2 and Sho1 are upstream sensors of the Ste50-Ste11-Ste7-Fmk1-Ste12 kinase cascade in *S. cerevisiae* and other fungi (Gu et al. 2015; Guo et al. 2017; Herrero de Dios et al. 2013; Rispail and Di Pietro 2010; Sharmeen et al. 2019; Takayama et al. 2019; Yamamoto et al. 2010). Here we compared the transcript levels of genes whose levels of transcription were reported to be regulated by the transcription factor *FoSte12* by quantitative real-time reverse transcriptase PCR (Gu et al. 2015). The results showed that the transcript levels of *FoOpy2* and three cellulase genes (*FOXG_14504; FOXG_13111; FOXG_16880*) were increased dramatically, but the transcript levels of other genes did not change significantly, in Δ*FoIme2* (Fig. 5). These results indicate that *Fo*Ime2 may act downstream of *Fo*Opy2 and function in parallel of *Fo*ste12. Deletion with *FoIme2* induced overexpress of *Fo*Opy2 through negative feedback regulation, and the overexpression *Fo*Opy2 increased the phosphorylation level of the downstream kinase Ste12, which eventually resulted in increased expression of two cellulase genes. The analysis agrees with what was reported in *U. maydis*, where the Ime2 homolog Crk1 is regulated by phosphorylation of both Fuz7 (the Ste7 homolog) and Kpp2 (the Fus3/Kss1 homolog), and it acts in the signal transduction pathway both in parallel to Kpp2 and as a substrate of this MAPK (Garrido et al. 2004). The process reported above is similar to what was shown in *T. reesei*, where the deletion of *Ime2* homologous genes results in significantly upregulated cellulose expression (Chen et al. 2015).

We also recorded a notable decrease (relative to WT) in the number of conidia in the *FoIme2*-mutants, however the mutation did not affect the conidial morphology. Our results are similar to the *Nrime2* mutant in *N. rileyi*: deletion of *Nrime2* delayed the sporulation time but did not affect conidial morphology (Li 2018). However, different results were reported in the case of the deletion of *AoIme2* in *A.ligospora*, the deletion of *Scime2* in *S. cerevisiae* and the deletion of *Sppit1* and *spmde3*, the two Ime2 homolog in *Schizosaccharomyces pombe*, which suggested that Ime2 pays an important role in conidial production and conidial morphology (Abe and Shimoda 2000; Schindler and Winter 2006; Xie et al. 2019).

The *FoIme2*-mutants displayed decreased resistance to extracellular osmotic (NaCl) stress and increased resistance to membrane (SDS) stress compared to the wild-type strain. However, no differences in tolerance to intracellular osmotic (KCl), cell wall (CR) and oxidative (H_2_O_2_) stress was identified. This is similar to the deletion of *AoIme2* in *A.ligospora*: *Aoime2* mutants were inhibited under hyperosmotic stresses but were unaffected by oxidative and cell-wall-perturbing stresses. In the case of *N. rileyi*, the *Nrime2* mutant displayed decreased growth rates after NaCl addition (Li 2018). Furthermore, the Ime2 homolog also plays a crucial role in environmental adaptation in *U. maydis* (Garrido and Pérez-Martín 2004). These results indicate that Ime2 is involved in the adaptation to high osmolarity stress in *F. oxysporum* and other fungi. In *S. cerevisiae*, the interaction between the transmembrane protein Opy2 and Sho1 enhances the signaling efficiency of the Hog1 MAP kinase cascade, which regulates the high osmolarity stress response (Takayama et al. 2019; Yamamoto et al. 2010). The different appearances of the *FoIme2*-mutants and the wild type in the presence of osmotic pressure may be caused by the *FoOPy2* overexpression in the mutants.

Despite the high expression levels of some cellulase genes, *Fo*Ime2 mutants displayed decreased virulence partly due to mycelial vegetative growth defects and decreased conidial yield. In the nematode-trapping fungi *A.ligospora, Aoime2* mutants displayed defects in capturing and infecting nematodes due to fewer traps, a fungal part that is essential to capture and infect nematodes. In *N. rileyi*, *Nrime2* deletion was also reported to severely affect virality (Li 2018), and *crk1* in *U. maydis* was required for pathogenicity (Garrido et al., 2004)*.* These results indicate that Ime2 plays a key role in fungal pathogenicity.

## Conclusions

In summary, our data indicate that the protein kinase *Fo*Ime2 plays a significant roles in the hyphal growth, hyphal branching, conidiation, stress response and pathogenicity of *F. oxysporum*. However, how *Fo*Ime2 exerts these functions, and the roles of *Fo*Ime2 in the MAPK system, require further study.

## Abbreviations

Fon: *Fusarium oxysporum* f.sp. *niveum*
Fon-1: *Fusarium oxysporum* f.sp. *niveum* race1
Ime2: The inducer of meiosis 2
MAPK: Mitogen-activated protein kinase
PDA: Potato dextrose agar medium
PDB: Potato glucose liquid medium dextrose broth
SNA: Synthetic low-nutrient agar medium
DI: Disease index
WT: Wild type stain
CR: Congo red

## References

[CR1] Abe H, Shimoda C (2000). Autoregulated expression of *Schizosaccharomyces pombe* meiosis-specific transcription factor Mei4 and a genome-wide search for its target genes. Genetics.

[CR2] Bayram O, Sari F, Braus GH, Irniger S (2009). The protein kinase ImeB is required for light-mediated inhibition of sexual development and for mycotoxin production in *Aspergillus nidulans*. Mol Microbiol.

[CR3] Chen F, Chen XZ, Su XY, Qin LN, Huang ZB, Tao Y, Dong ZY (2015). An Ime2-like mitogen-activated protein kinase is involved in cellulase expression in the filamentous fungus *Trichoderma reesei*. Biotechnol Lett.

[CR4] Di Pietro A, Roncero MI (1998). Cloning, expression, and role in pathogenicity of pg1 encoding the major extracellular endopolygalacturonase of the vascular wilt pathogen *Fusarium oxysporum*. Mol Plant Microbe Interact.

[CR5] Di Pietro A, Garcia-MacEira FI, Meglecz E, Roncero MI (2001). A MAP kinase of the vascular wilt fungus *Fusarium oxysporum* is essential for root penetration and pathogenesis. Mol Microbiol.

[CR6] Fulton JC, Cullen MA, Beckham K, Sanchez T, Xu ZX, Stern P, Vallad G, Meru G, McGregor C, Dufault NS (2021). A contrast of three inoculation techniques used to determine the race of unknown *Fusarium oxysporum* f.sp. niveum Isolates. Jove-J Visual Exp.

[CR7] Garrido E, Voss U, Muller P, Castillo-Lluva S, Kahmann R, Perez-Martin J (2004). The induction of sexual development and virulence in the smut fungus *Ustilago maydis* depends on Crk1, a novel MAPK protein. Genes Dev.

[CR8] Gu Q, Zhang C, Liu X, Ma Z (2015). A transcription factor FgSte12 is required for pathogenicity in *Fusarium graminearum*. Mol Plant Pathol.

[CR9] Guo N, Qian Y, Zhang Q, Chen X, Zeng G, Zhang X, Mi W, Xu C, St Leger RJ, Fang W (2017). Alternative transcription start site selection in Mr-OPY2 controls lifestyle transitions in the fungus *Metarhizium robertsii*. Nat Commun.

[CR10] Herrero de Dios C, Roman E, Diez C, Alonso-Monge R, Pla J (2013). The transmembrane protein Opy2 mediates activation of the Cek1 MAP kinase in *Candida albicans*. Fungal Genet Biol.

[CR11] Hudson O, Waliullah S, Fulton JC, Ji PS, Dufault NS, Keinath A, Ali ME (2021). Marker development for differentiation of *Fusarium oxysporum* f.sp. *Niveum* Race 3 from Races 1 and 2. Int J Mol Sci.

[CR12] Hutchison EA, Glass NL (2010). Meiotic regulators Ndt80 and ime2 have different roles in *Saccharomyces* and *Neurospora*. Genetics.

[CR13] Hutchison EA, Bueche JA, Glass NL (2012). Diversification of a protein kinase cascade: IME-2 is involved in nonself recognition and programmed cell death in *Neurospora crassa*. Genetics.

[CR14] Irniger S (2011). The Ime2 protein kinase family in fungi: more duties than just meiosis. Mol Microbiol.

[CR15] Kou Y, Naqvi NI (2016). Surface sensing and signaling networks in plant pathogenic fungi. Semin Cell Dev Biol.

[CR16] Li C (2018) The gene cloning and functional analysis of Rim15, Ume6 and Ime2 Gene in *Nomuraea Rileyi*. [dissertation/master’s thesis]. Chongqing: ChongqingUniversity

[CR17] Liu KH, Shen WC (2011). Mating differentiation in *Cryptococcus neoformans* is negatively regulated by the Crk1 protein kinase. Fungal Genet Biol.

[CR18] Livak KJ, Schmittgen TD (2001). Analysis of relative gene expression data using real-time quantitative PCR and the 2(-Delta Delta C(T)) Method. Methods.

[CR19] Martinez-Soto D, Ruiz-Herrera J (2017). Functional analysis of the MAPK pathways in fungi. Rev Iberoam Micol.

[CR20] Nguyen TV, Tran-Nguyen LTT, Wright CL, Trevorrow P, Grice K (2019). Evaluation of the efficacy of commercial disinfectants against *Fusarium oxysporum* f.sp. *cubense* Race 1 and Tropical Race 4 Propagules. Plant Dis.

[CR21] Pareek M, Rajam MV (2017). RNAi-mediated silencing of MAP kinase signalling genes (Fmk1, Hog1, and Pbs2) in *Fusarium oxysporum* reduces pathogenesis on tomato plants. Fungal Biol.

[CR22] Rispail N, Di Pietro A (2010). The homeodomain transcription factor Ste12: connecting fungal MAPK signalling to plant pathogenicity. Commun Integr Biol.

[CR23] Sari F, Heinrich M, Meyer W, Braus GH, Irniger S (2008). The C-terminal region of the meiosis-specific protein kinase Ime2 mediates protein instability and is required for normal spore formation in budding yeast. J Mol Biol.

[CR24] Schindler K, Winter E (2006). Phosphorylation of Ime2 regulates meiotic progression in *Saccharomyces cerevisiae*. J Biol Chem.

[CR25] Segorbe D, Di Pietro A, Perez-Nadales E, Turra D (2017). Three *Fusarium oxysporum* mitogen-activated protein kinases (MAPKs) have distinct and complementary roles in stress adaptation and cross-kingdom pathogenicity. Mol Plant Pathol.

[CR26] Sharmeen N, Sulea T, Whiteway M, Wu C (2019). The adaptor protein Ste50 directly modulates yeast MAPK signaling specificity through differential connections of its RA domain. Mol Biol Cell.

[CR27] Smith HE, Mitchell AP (1989). A transcriptional cascade governs entry into meiosis in *Saccharomyces cerevisiae*. Mol Cell Biol.

[CR28] Strudwick N, Brown M, Parmar VM, Schroder M (2010). Ime1 and Ime2 are required for pseudohyphal growth of *Saccharomyces cerevisiae* on nonfermentable carbon sources. Mol Cell Biol.

[CR29] Takayama T, Yamamoto K, Saito H, Tatebayashi K (2019). Interaction between the transmembrane domains of Sho1 and Opy2 enhances the signaling efficiency of the Hog1 MAP kinase cascade in *Saccharomyces cerevisiae*. PLoS One.

[CR30] Turra D, Segorbe D, Di Pietro A (2014). Protein kinases in plant-pathogenic fungi: conserved regulators of infection. Annu Rev Phytopathol.

[CR31] Vandermeulen MD, Cullen PJ (2020). New aspects of invasive growth regulation identified by functional profiling of MAPK pathway targets in *Saccharomyces cerevisiae*. Genetics.

[CR32] Wu PH, Ho YL, Ho TS, Chang CH, Ye JC, Wang CH, Sung HM, Huang HJ, Liu CC (2019). Microbial volatile compounds-induced cytotoxicity in the yeast *Saccharomyces cerevisiae*: the role of MAPK signaling and proteasome regulatory pathway. Chemosphere.

[CR33] Xie M, Bai N, Yang J, Jiang K, Zhou D, Zhao Y, Li D, Niu X, Zhang KQ, Yang J (2019). Protein Kinase Ime2 is required for mycelial growth, conidiation, osmoregulation, and pathogenicity in nematode-trapping fungus *Arthrobotrys oligospora*. Front Microbiol.

[CR34] Yamamoto K, Tatebayashi K, Tanaka K, Saito H (2010). Dynamic control of yeast MAP kinase network by induced association and dissociation between the Ste50 scaffold and the Opy2 membrane anchor. Mol Cell.

